# A retrospective study of risk factors for poor outcomes in methicillin-resistant staphylococcus aureus (MRSA) infection in surgical patients

**DOI:** 10.1186/1749-799X-6-25

**Published:** 2011-05-23

**Authors:** Kelechi C Eseonu, Scott D Middleton, Chinyere C Eseonu

**Affiliations:** 1 Orthopaedic Trauma Unit, Royal Infirmary, Edinburgh, EH16 4SA, UK; 2 University College London Medical School, Gower Street, London, WC1E 6NT, UK

## Abstract

**Background:**

Since its isolation, Methicillin-resistant Staphlococcus aureus (MRSA) has become a major cause of hospital acquired infection (HAI), adverse patient outcome and overall resource utilisation. It is endemic in Scotland and widespread in Western hospitals. MRSA has been the subject of widespread media interest- a manifestation of concerns about sterile surgical techniques and hospital cleanliness. This study aimed to investigate patient outcome of MRSA infections over the last decade at a major orthopaedic trauma centre. The objective was to establish the association of variables, such as patient age and inpatient residence, against patient outcome, in order to quantify significant relationships; facilitating the evaluation of management strategies with an aim to improving patient outcomes and targeting high-risk procedures.

**Methods:**

This is a retrospective study of the rates and outcomes of MRSA infection in orthopaedic trauma at the Royal Infirmary of Edinburgh. Data was collated using SPSS 14.0 for Windows(R). Shapiro-Wilkes testing was performed to investigate the normality of continuous data sets (e.g: age). Data was analysed using both Chi-Squared and Fisher's exact tests (in cases of expected values under 5)

**Results:**

This study found significant associations between adverse patient outcome (persistent deep infection, osteomyelitis, the necessity for revision surgery, amputation and mortality) and the following patient variables: Length of inpatient stay, immuno-compromise, pre-admission residence in an institutional setting (such as a residential nursing home) and the number of antibiotics used in patient care. Despite 63% of all infections sampled resulting from proximal femoral fractures, no association between patient outcome and site of infection or diagnosis was found. Somewhat surprisingly, the relationship between age and outcome of infection was not proved to be significant, contradicting previous studies suggesting a statistical association. Antibiotic prophylaxis, previously identified as a factor in reducing overall incidence of MRSA infection, was not found to be significantly associated with outcome.

**Conclusions:**

Early identification of high-risk patients as identified by this study could lead to more judicious use of therapeutic antibiotics and reductions in adverse outcome, as well as socioeconomic cost. These results could assist in more accurate risk stratification based on evidence based evaluation of the significance of the risk factors investigated.

## Introduction

The results of surveillance of 41,242 operations for surgical site infections in orthopaedic surgery (SSIS), (April 2007 to March 2008), showed that 48% of SSIs were caused by Staph. Aureus, of which 68% were MRSA [1]. As of early 2007, the number of deaths in the United Kingdom attributed to MRSA was estimated to be around 3,000 annually [2].

Research on MRSA has tended to concentrate on epidemiology, rather than outcomes. The cost of orthopaedic infection is considerable, with a retrospective study, conducted by a single District General Hospital in 2008 estimating the annual cost of MRSA infection in its' orthopaedic setting to be almost £390,000 [3].

Despite debate as to the virulence of methicillin-resistant Staphylococcus aureus (MRSA) when compared with methicillin-susceptible S. aureus (MSSA), rates of mortality of MRSA bacteraemia are thought to be higher than those associated with MSSA [4].

## Methods

This study is a retrospective review of admissions over an 11 year period from 1^st ^March 1999 in the Trauma Department of Orthopaedic Surgery at the Royal Infirmary of Edinburgh. Over this period, there were 37960 'trauma' (emergency, non elective) admissions to the unit requiring surgical intervention. Of these, there were 404 MRSA post-operative wound infections and an additional 254 patients were noted as being 'colonised' by MRSA. Overall incidence of MRSA wound infection over this period was 1.06%. Our randomised sample included 15% of all cases over this period. Patient details were retrospectively collated from an orthopaedic database for name, date of birth, gender, immunocompromise, diabetes, pre-admission residence (home or institutional setting), diagnosis, time from injury to procedure, use of arthroplasty, length of inpatient residence, number of antibiotics used, concomitant surgical site infection (SSI), number of revision procedures and site of post-surgical infection. Additional note was taken if therapeutic serum Vancomycin levels had been monitored.

### Definitions

**Figure 1:**
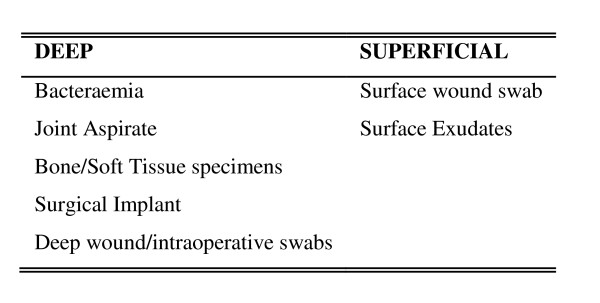
**Classification of MRSA Infections/Colonisation**.

Positively identified MRSA cases were classified as superficial or deep with respect to the location of the specimen site [see Figure 1].

### Data Collection and Statistical Analysis

**Figure 2:**
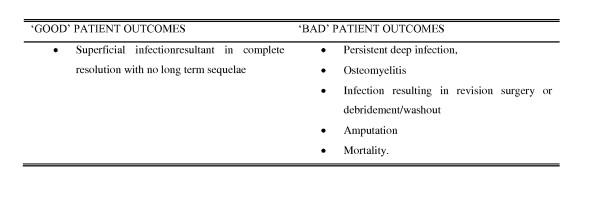
**Classification of post operative clinical outcomes**.

**Figure 3:**
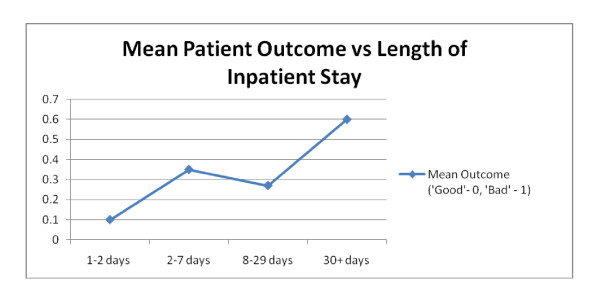
**Patient outcome against Patient Variable (Length of In-patient stay)**.

Cases were imported to SPSS 14.0 for Windows^®^. Data was analysed using Chi-Squared testing. Patients were grouped into categories for *'age' *and *'time from original injury'*. Values are given to three significant figures, except for p-values, which are given to two decimal places. Patient outcome was assessed for significance (p < 0.05) and strength of association (using Cramer's V values). We utilised a simple binary system, categorising a 'good' outcome (e.g. discharge without complication) as a '0' and an adverse outcome (e.g. necessary revision surgery due to deep post operative infection) as a '1' [see Figure 2]. This allowed us to calculate mean post operative outcomes, which we subsequently compared to a number of patient variables and co-morbidities [see Figure 3].

## Results

We identified a randomised sample of 61 orthopaedic trauma admissions over the period January 1998 to March 2009. 59% of patients experienced 'good' outcomes to their infections, whilst 41% suffered 'adverse' outcomes (definitions above). Associations between variables and patient outcome were investigated at the 95% significance level (p < 0.05)

## Risk Factors

**Figure 4:**
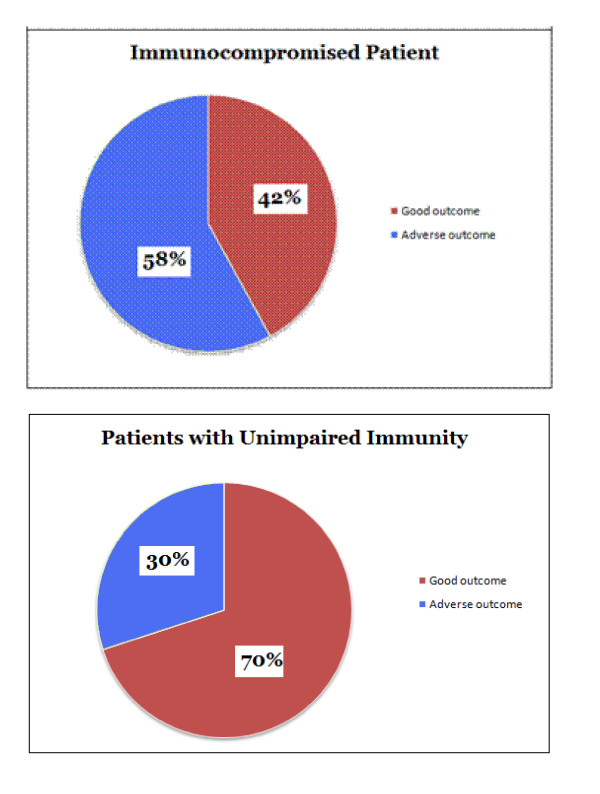
**Immuno-compromise against patient outcome**.

We demonstrated a significant association between patient immuno-compromis**e **and adverse outcome **(x**^**2**^**= 4.92 p = 0.026)**. 58% of immuno-compromised patients had adverse outcomes, compared to 30% of patients without impaired immunity. This relationship was significant, but of a moderate strength **(Cramer's V: 0.284) **[Figure 4].

**Figure 5:**
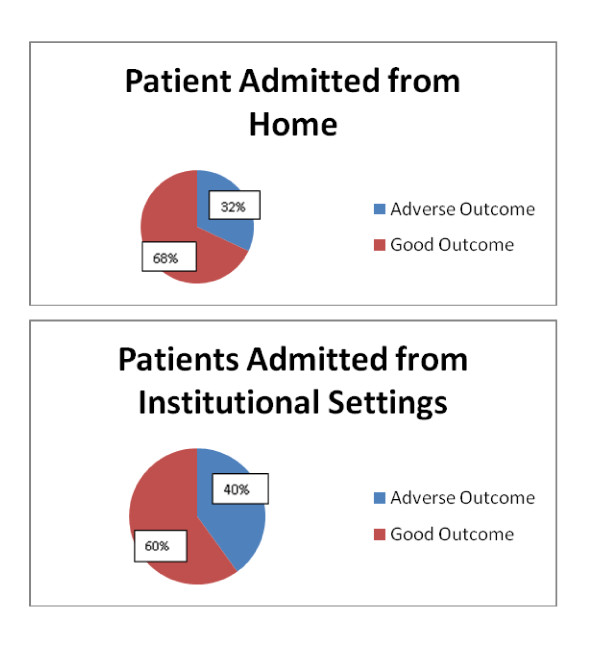
**Preadmission residence against patient outcome**.

**Pre-admission residence **is a well documented factor in MRSA incidence and a significant association with patient outcome was shown. **(x**^2^**= 4.45, p = 0.035)**. 32% of patients admitted from home had adverse outcomes, compared to 40% of patients admitted from an institutional setting, such as a nursing home or another hospital ward. [Figure 5]. This association was significant, even when randomising for the high mean age of patients admitted from institutional settings. **(x**^2^**= 3.75, p = 0.045 Cramer V = 0.394)**. The latter had a risk ratio (RR) of 1.25 of experiencing adverse outcomes when compared to patients admitted from home.

**Length of Inpatient residence (LOS) **was found to be significantly associated with adverse outcome. **(x**^**2**^**= 8.87, p = 0.03 Cramer V = 0.458)**. This association was the strongest of all the variables tested [Figure 3]. 62% of patients with an LOS greater than 30 days suffered adverse outcomes compared to 24% of patients with an LOS less than 30 days. The distribution of LOS in MRSA patients was positively skewed against normality with a median LOS of 27 days compared to 4 days of inpatient stay in the non-MRSA population in the same unit [5].

## Negative Results

### Gender

**Figure 6:**
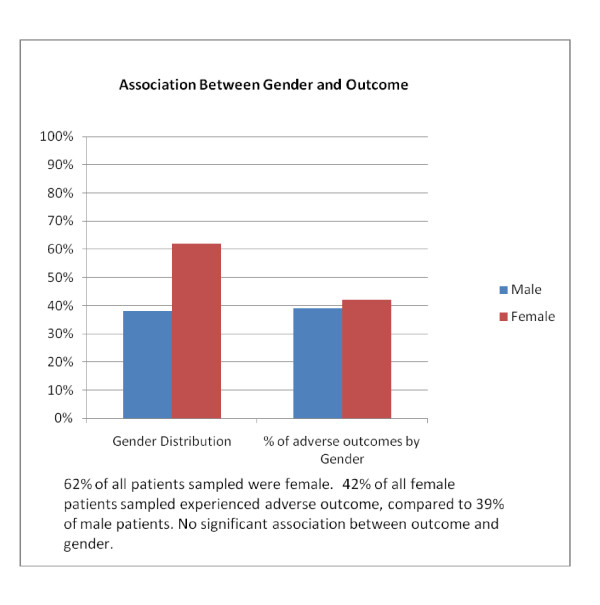
**Gender against patient outcome**.

Past studies have suggesting a higher incidence of post-operative MRSA infections in males [4]. 38% of our cohort was male and 62% female. We found no significant relationship between gender and outcome **(x**^**2**^**= 0.52, p-value = 0.819)**. The predominance of elderly patients in orthopaedic trauma is well established. Whilst 72% of our cohort were over the age of 65 on admission, **(mean age: 70.2 yrs) **29% of patients under-65's were female, compared to 75% over the age of 65. This perhaps relates to behavioural patterns and incidence of traumatic injuries through risk taking behaviours amongst younger men, as well as the rates of osteoporosis and cortical degeneration in older women. [Figure 6].

The mean age of the cohort with a 'good' outcome was 71, while the mean age of the **'adverse' **outcome subset was 69. Contrary to the findings of previous work, we found no significant association. **(x**^2^**= 0.001 p = 0.985) **[6].

No significant relationship was found between **antibiotic prophylaxis **and outcome **(x**^2^**= 8.80; p = 0.348)**. Indeed, 36% of those who were not given prophylactic antibiotics had an adverse outcome, compared to 44% of those who did receive prophylaxis. However, older patients appeared more likely to receive prophylaxis than younger patients and also were also more likely to have their daily serum Vancomycin levels monitored on a more frequent basis. However, this association was not significant. **(x**^2^**= 3.42 p = 0.064)**

There was no significant association between diabetes or arthroplasty use and outcome **(x**^2^**= 1.36 p = 0.730)**. 40% of non-diabetics and 50% of diabetics suffered adverse outcomes, but this association was not significant **(Fisher's exact test p-value 0.642)**. No significant association was found between **time from injury to procedure **and patient outcome. **(Fisher's exact p-value 0.823)**

**Figure 7:**
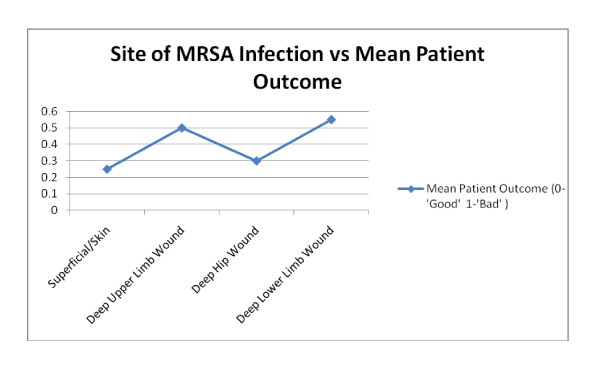
**Infection Site against patient outcome**.

No significant association was found between **site of infection **and patient outcome **(Fisher's p value 0.562)**. Superficial wound infection was found to be associated with best mean outcomes, while neck of femur (NOF) fracture wounds and other lower limb wounds were associated with worse outcomes [Figure 7]. 63% of all cases involved extracapsular and intracapsular hip fractures. 68% of these cases were in females and 89% of these cases were in patients over the age of 65. Overall, 37% of intracapsular and extracapsular hip fractures were linked to adverse outcomes.

Despite the high frequency of MRSA infection associated with proximal neck of femur fractures, especially in the elderly, no significant association was found between **diagnosis **and the outcome of infection. **(x**^2^**= 3.63 p = 0.459) **[4].

## Discussion

The increasing incidence of MRSA colonisation in patients from institutional settings is well documented and rates of nosocomial MRSA infection have increased over the past decade according to numerous studies [5,7]. However, data on the effect of relevant variables on mortality, (rather than epidemiology) is more sparse. Post-operative MRSA infection stabilised in 2006, with the number of UK MRSA related deaths peaking at 1652 in 2006, up from 51 in 1993. Changes in reporting practices comprise a proportion of this change, but an upward trend is still apparent.

## Associations with Patient Outcome

### Site of Pre-Admission Residence

This is particularly significant, given the high mean age and proportion of patients from institutional settings. Interestingly, heterogeneity *between *institutional settings is noted. Data from the Office of National Statistics showed a Risk Ratio (RR) of 8.0 for MRSA colonisation in patients from NHS nursing homes, compared to patients from private care homes. Much of the evidence for United Kingdom guidelines for MRSA prevention in healthcare facilities was generated in acute care settings and may not be directly transferable to the nursing home environment. However, the incidence of colonisation in residential patients is comparable to that of hospitalised populations and patients transferred from long-term care facilities to hospital often act as nosocomial reservoirs of MRSA [5,6]. Additionally, there is a suggestion of possible latent acquisition of deep post operative wound infection from colonisation as a result of prior repeated exposures to healthcare facilities and residential settings which could explain this trend [8]. This conclusion reinforces the importance of preventing initial MRSA colonisation in this high risk group by judicious use of prophylactic antibiotic therapy.

### Duration of Inpatient Stay (LOS)

The association between LOS and staphylococcal infection is well substantiated. Research has demonstrated that MRSA infected patients suffer increased length of hospitalisation when compared to uninfected patients [9]. Evidence has identified healthcare workers as possible reservoirs for nasal colonisation a factor known to predispose to increased risk of post-operative wound infection, especially in the elderly [10,11]. In the UK, the most common strains of MRSA are EMRSA15 and EMRSA16 [12]. The latter has been particularly successful in developing resistance to erythromycin and ciprofloxacin and surviving intracellularly and is thought to be more prevalent in healthcare workers than the general population [13].

There is a suggestion that MRSA infection impairs post operative wound healing and it is unclear whether the association with LOS is a cause or result of infection [14]. Further investigation could monitor LOS *before *initial isolation of a MRSA, but there are difficulties in identifying the exact onset of wound infection. Patients from the poorest socio-economic backgrounds are reportedly up to seven times more likely to get postoperative infection with MRSA than more affluent social groups, possibly reflecting frequency of hospital admissions, rather than CA-MRSA infection [15]. Further study of individuals with frequent inpatient admissions and the outcomes of any subsequent MRSA infection could result in better screening of such individuals.

## Number of antibiotics used and monitored serum Vancomycin

A variety of studies have suggested that antibiotic exposure may be a risk factor of MRSA isolation but the association with mortality is less well defined [16]. One study in particular highlighted a 1.8 fold increase in MRSA isolation in patients prescribed more than 2 antibiotics in the last 180 days [16]. Clearly, patients with more perceivably dire clinical prognosis could be managed by more antibiotics and it is unclear whether this association is a cause or a result of a developing outcome.

Interestingly, monitoring serum vancomycin levels was not found to be linked to positive outcomes. Studies have shown that the empirical use of Vancomycin may not be judicious in MRSA and may increase mortality, especially when responsible strains have a high vancomycin MIC (minimum inhibitory concentration). Even when MRSA is susceptible to vancomycin [MIC ≤ 2 μg/mL], in treating MRSA bacteraemia is not unusual, due to changes in the MIC or heteroresistance. For patients with sepsis in MRSA bacteraemia, appropriate selection of empirical antimicrobial treatment has been shown to be a major prognostic factor [16]. In these cases, newer anti-staphylococcal agents, such as linezolid and daptomycin could be superior to vancomyin [15,17].

## Immunocompromised Patients and Diabetes

Research on the impact of immunocompromise on MRSA outcome is surprisingly scant. Studies regarding patients with upper thoracic cancers have linked MRSA infection (40%) in post-operative patients with significant morbidity [3]. A study has suggested a link between HIV and community acquired MRSA(CA-MRSA). It highlighted a 2-fold increase in adverse outcomes in immunocompromised patients, a conclusion broadly supported by our study [18].

Our results were not sufficiently statistically significant to support an association between diabetes and clinical outcome. (Past studies suggest an association between diabetes and SSI's) [19]. Our results may have been hampered by our sample size, but our validity was improved by correction for the high mean age of patients with diabetes and MRSA isolation.

## Other Interesting and Negative Findings

No significant association was found between age and outcome. This contradicts research suggesting an increase in mortality with age in MRSA patients. (A recent study suggested an odds ratio of mortality of 2.74 (95% confidence interval) for >75 compared with ≤60 yr old patients) [3]. The distribution of age in MRSA infection in our sample was heavily positively skewed. As a result, our small sample size resulted in a low number of patients below the age of 65, reducing the significance of our results in this subset.

## Conclusion

*This study highlights associations between outcome and immunocompromise, length of inpatient stay and pre-admission residence, which are significant and substantiated by past studies. These conclusions suggest that targeted MRSA prophylaxis should be offered to high risk patients identified by appropriate risk stratified techniques, based on the risk factors noted in results. My literature review has shown the overall scarcity of literature related to outcome of MRSA infection and in the context of a wealth of information regarding the epidemiology, more comprehensive research is needed*.

## Competing interests

The authors declare that they have no competing interests.

## Authors' contributions

KE conceived the study, participated in data collection and analysis, drafted the manuscript and coordinated the study. SM participated in statistical analysis, creation of figures and tables and addressing the corrections. CE participated in study design and drafting of the manuscript. All authors read and approved the final manuscript.

## References

[B1] Guyot A Layer G MRSA - 'bug-bear' of a surgical practice: reducing the incidence of MRSA surgical site infections Annals of the Royal College of Surgeons of England 2006 88 2 222 3 10.1308/003588406X94841 16551425 PMC1964055

[B2] Gray JW George RH Is the incidence of MRSA bacteraemia representative of the rate of MRSA infection in general? Journal of Hospital Infection 2001 49 1 79 10.1053/jhin.2001.1041 11516192

[B3] Wyllie DH Crook DW Peto TE Mortality after Staphylococcus aureus bacteraemia in two hospitals in Oxfordshire Cohort Study. British Medical Journal 1997 333 7562 281 10.1136/bmj.38834.421713.2F PMC1526943 16798756

[B4] Gould IM Costs of hospital-acquired methicillin-resistant Staphylococcus aureus (MRSA) and its control International Journal of Antimicrobial Agents 2006 28 5 379 84 10.1016/j.ijantimicag.2006.09.001 17045462

[B5] Menon KV Whiteley MS Burden P Galland RB Surgical patients with methicillin resistant staphylococcus aureus infection: an analysis of outcome using P-POSSUM JR Coll Surg Edinb 1999 44 161 3 10372484

[B6] Hughes CM Smith MB Tunney MM Infection control strategies for preventing the transmission of methicillin-resistant Staphylococcus aureus (MRSA) in nursing homes for older people Cochrane Database Syst Rev 2008 1 CD006354 10.1002/14651858.CD006354.pub2 18254100

[B7] Stefani S Varaldo PE Epidemiology of methicillin-resistant staphylococci in Europe Clin Microbiol Infect 2003 9 12 1179 86 10.1111/j.1469-0691.2003.00698.x 14686982

[B8] Revised guidelines for the control of Methicillin-resistant Staphylococcus aureus in hospitals J Hosp Infect 2006 39 4 British Society for anti-microbial Chemotherapy, hospital Infection society and the Infection Control Nurses Association 10.1016/s0195-6701(98)90293-6 9749399

[B9] Cosgrove SE Qi Y Kaye KS Harbarth S Karchmer AW Carmeli Y The impact of Methicillin Resistance in Staphylococcus aureus Bacteremia on Patient Outcomes: Mortality, Length of Stay, and Hospital Charges Infection Control and Hospital Epidemiology 26 166 174 10.1086/502522 15756888

[B10] Rahij Anwar Rajesh Botchu Manoj Viegas Preoperative methicillin-resistant Staphylococcus aureus (MRSA) screening: An effective method to control MRSA infections on elective orthopaedics wards Surgical Practice 2006 10 4 135 137 10.1111/j.1744-1633.2006.00314.x

[B11] Wenzel RP Perl TM The significance of nasal carriage of *Staphylococcus aureus *and the incidence of postoperative wound infection Journal of Hospital Infection 1995 31 1 13 24 10.1016/0195-6701(95)90079-9 7499817

[B12] Holden MTG Feil EJ Lindsay JA "Complete genomes of two clinical Staphylococcus aureus strains: Evidence for the rapid evolution of virulence and drug resistance" Proc Natl Acad Sci USA 2004 101 9786 91 10.1073/pnas.0402521101 15213324 PMC470752

[B13] Johnson AP Aucken HM Cavendish S "Dominance of EMRSA-15 and -16 among MRSA causing nosocomial bacteraemia in the UK: analysis of isolates from the European Antimicrobial Resistance Surveillance System (EARSS)" J Antimicrob Chemother 2001 48 1 143 4 10.1093/jac/48.1.143 11418528

[B14] Dissemond J Practical consequences after MRSA identification in chronic wounds Hautarzt 2007 58 11 952 8 10.1007/s00105-007-1403-0 17926013

[B15] Hazzan R Paul M Shaked H Effect of Adequate empiric Antibiotic therapy on the survival of patients with MRSA Journal of Thrombosis and Haemostasis 2007 5 1

[B16] Alex Soriano Francesc Marco Martinez JA "Influence of Vancomycin Minimum Inhibitory Concentration on the Treatment of Methicillin-Resistant Staphylococcus aureus Bacteremia" Clinical Infectious Diseases 2008 46 193 200 2007 10.1086/524667 18171250

[B17] Bootsma MC Diekmann O Bonten MJ Controlling Methicillin-resistant Staphylococcus Aureus: Quantifying the effects of interventions and rapid diagnostic testing Proc Natl Acad Sci USA 2006 103 5620 5 10.1073/pnas.0510077103 16565219 PMC1459403

[B18] Venditti M Falcone M Micozzi A Staphylococcus aureus bacteremia in patients with hematologic malignancies: a retrospective case control study Haematologica 88 8 923 30 12935981

[B19] Subbe CP Rao GG Sedgwick P Van Heerden N Groba CB MRSA: predictor of outcome in critically ill patients British Journal of Anaesthesia 2000 84 1 662 662 Number 5

